# Elimination of cervical cancer: challenges for developing countries

**DOI:** 10.3332/ecancer.2019.975

**Published:** 2019-11-12

**Authors:** Ann Marie Beddoe

**Affiliations:** Department of Obstetrics, Gynecology and Reproductive Science, Icahn School of Medicine at Mount Sinai, New York, NY 10029, USA

**Keywords:** human papillomavirus (HPV) vaccination, cervical cancer, prevention, screening, disparities in care, visual inspection with acetic acid, HPV testing

## Abstract

Cervical cancer is one of the most preventable cancers today, yet over 500,000 new cases are diagnosed globally each year and every 2 minutes a woman dies from cervical cancer. The burden of this disease and the highest mortality from cervical cancer occur in developing countries. High-income countries are poised to eliminate cervical cancer in the 21st century, but despite a global call to eliminate this disease, low- and middle-income countries face many challenges as they strive to answer that call.

Cervical cancer is the fourth most common cancer in women worldwide and a major cause of cancer-related mortality. Approximately 570,000 new cases and 311,365 deaths were estimated in 2018 [1]. More than any other cancer, cervical cancer highlights the great disparities in incidence and mortality that exist between developed and developing countries. Over 85% of new cases and 80% of mortality from cervical cancer occur in low- and middle-income countries (LMICs) [2]. In contrast, high-income countries (HICs) have seen a steady decline in both incidence and mortality, attributed to well-organised screening programmes and infrastructure to provide appropriate follow-up and treatment. Due to effective screening and treatment in the United States (US), only 13,170 new cases of cervical cancer and 4,250 deaths are expected to occur in 2019 [3, 4]. In the backdrop of this success in managing cervical cancer in the developed world, the WHO has called for the worldwide elimination of cervical cancer through a global call to action made by United Nations (UN) Director, General Dr T. A. Ghebreyesus [5]. While total eradication of cervical cancer is within the reach of the most developed countries, eradication is more challenging in the developing world. Thus, the question remains as to whether developing countries will again be left behind as these goals are pursued.

The current state of cervical cancer in developing countries in many ways parallels cervical cancer in the inner city US populations 40 years ago. During my residency/fellowship in obstetrics and gynecology and gynecologic oncology between 1978 and 1982, the most common cancer treated at Kings County Hospital-SUNY Downstate Medical Center (Brooklyn, NY, USA) was cervical cancer. It was a time when the majority of women diagnosed with cervical cancer had never had a Papanicolau (Pap) smear [6], and when we hypothesised based on studies showing low cervical cancer incidence in nuns, that cervical cancer was, indeed, associated with a sexually transmitted infection (STI) [7]. At that time, multiple STIs including chlamydia, herpes virus and human papillomavirus (HPV) were all implicated in the development of cervical dysplasia, but the role of HPV, the specific agent that is causally linked to cervical cancer, had not been elucidated [8]. What was present in the US at that time were basic screening tools: availability of Pap smears, colposcopy, pathology infrastructure, treatment facilities and organised cancer registration. This infrastructure allowed us as a country to better organise our screening programmes over time and later incorporate advancing technology to drive down the incidence of cervical cancer, positioning us to achieve the goal of eliminating cervical cancer well within the 21st century.

Unfortunately in most countries with inadequate health and social systems, these basic tools are lacking, and compounded with limited cervical cancer awareness and education, elimination of this disease is a major challenge [9]. In Sub-Saharan Africa (SSA), although incidence rates vary from country to country, approximately 93,225 new cases of cervical cancer occurred in 2012, making it the second most common cancer of reproductive-aged women in the region [10]. South Africa and East Africa share the highest burden of cervical cancer, where age-standardised incidence rates of 43.1 and 40.1 per 100,000, respectively, exist [11]. The highest incidence of cervical cancer in SSA is, however, found in Malawi where age-standardised incidence rates of >72.9 per 100,000 and mortality rates of 49.8 per 100,000 from this disease occur [12]. In 2004, the Malawian Ministry of Health-Reproductive Health Directorate (MoH-RHD) introduced a comprehensive cervical cancer screening programme using visual inspection with acetic acid (VIA) and cryotherapy following a gradual scaling up of activities that began in the 1980s. The goal of this programme was to screen 80% of Malawi’s eligible women. Despite the programme falling short of its goals, the percentage of women screened did increase from 9.3% to 26.5%, demonstrating some measure of success, but with major challenges faced in ‘lost to follow-up’ of positive patients and equipment failure when patients were recalled [13]. Maseko *et al* [14] reported on additional challenges that the two-decade cervical cancer programme faced, focusing on national policy rather than the more commonly assessed cultural and socioeconomic barriers that plague most developing countries’ cancer control efforts. Their research suggested that because cervical cancer control was a sub-theme of a broader Health Sector Strategic Plan (HSSP) that focused on general reproductive care, it did not specifically address those issues unique to cervical cancer prevention and control such as type of screening, target populations to be screened, frequency of screening or target ages and did not include any information on HPV vaccinations. They also looked at non-policy challenges to decentralised screening programmes and found that health workforce readiness and training, as well as lack of equipment and supplies, were also contributing factors [15].

In Nigeria, the most populous country in Africa, almost 15,000 new cases of cervical cancer are diagnosed annually and the age-standardised incidence rate is 27.1 per 100,000 [16]. Nigeria has developed a National Cancer Control Policy that incorporates cervical cancer awareness, implementation of HPV-DNA testing/VIA with the treatment of pre-cervical lesions and implementation of HPV vaccination programmes [17]. Currently, HPV vaccination is available on a private basis only, but efforts are ongoing to incorporate HPV vaccination into their routine immunisation programmes. Although this policy has been proposed, due to its cost, the feasibility for implementation and sustainability on a national level seems unlikely given Nigeria’s population of >200M people, 50M of whom are females over the age of 15 [18]. An analysis of various cervical cancer screening scenarios conducted by Ekwunife and Lhachimi using Nigerian specific modelling data concluded that opportunistic cervical cancer screening with VIA combined with national HPV vaccination was the most cost-effective method of cervical cancer screening in Nigeria. Although this method was the least costly, the authors concluded that a 34% reduction in cervical cancer could be achieved, but based on Nigeria’s current economic constraints, implementation of this programme would be challenging [19].

Like Nigeria, many developing countries will have to wrestle with the cost of national cervical cancer elimination strategies. There are, however, lessons to be learned that will bring developing nations within reach. Strong governmental support and buy-in are essential in promoting and implementing education, prevention, screening, diagnostic, treatment and terminal care capacity. Policy must be implemented within the geographic and cultural context of each particular country so that both rural and urban communities have access to affordable care such that women living in the least accessed regions can be equally served [20]. With the introduction of the HPV vaccine in 2006, attributed to Ian Frazer and Jian Zhou and based on Zur Hausen’s work in the 1970s, primary prevention is now possible and paramount in the fight against cervical cancer. The impact of the vaccine on cervical cancer prevention, however, depends on having high vaccine coverage of the target population in order to achieve sustainable cross-protection and immunity [21].

The first country to successfully introduce large-scale HPV vaccination was Australia. This publicly funded programme, which began in 2007, administered primary vaccination to adolescent girls aged 12–13 years. For females between the ages of 18 and 26 who were not previously vaccinated, the programme also offered free ‘catch-up’ vaccinations. Combined with a robust screening programme that included both transitioning from cytology to HPV testing in 2017, and a high vaccination coverage (80% for females), Australia appears to be one of the first countries on track to eliminate cervical cancer. Assuming vaccination and screening rates remain high and using a model that includes HPV vaccination, natural history and screening, their elimination goal could be realised by 2028 based on a threshold of four cases per 100,000 [22]. As posited by Baussano and Bray, Australia’s projection was based on a data generated modelling system that most developed countries could utilise as they attempt to eliminate cervical cancer [23]. For most low-income countries (LICs), however, data collection and reporting are very limited. Therefore, a modelling system that depends on accurate country data may not be the best predictor of cervical cancer elimination in these low-resource settings. These authors have suggested that a shift towards building data collection and analysis capacity may play a larger role in helping LMICs strategise local cancer control and policy development to better target their elimination goals [23].

Utilising a completely different strategy, Rwanda, despite lacking a cancer registry or cancer policy, became the first African country to implement a national prevention programme for cervical cancer in 2013 [24]. Several pre-immunization activities that included establishing a diverse technical group with sub-committees that managed detailed immunization logistics, helped the HPV vaccination campaign to be successful [25]. Community health workers and nurses spent several weeks promoting it as a cancer prevention vaccine. They took time to dispel myths associated with the HPV vaccine. Moreover, spiritual leaders, government officials, teachers and volunteers supported educational programmes, direct person-to-person and billboard advertising. Rwanda has surpassed Australia in HPV vaccination coverage reaching >93% of girls aged 11–15 years old [10]. Simultaneously, Rwanda has initiated national cervical cancer screening using visual inspection with acetic acid (VIA), as well as breast cancer screening using clinical breast exam (CBE).

The Australian strategy relies heavily on sustained broad coverage for both screening and vaccine-uptake and continued economic support from the government. The already built-in infrastructure that provides histologic diagnosis and therapy for patients who have existing cervical cancer make a short-term goal of elimination realistic. However, Johnson *et al* [26] raised one potential unintended consequence of further increasing cervical cancer disparities using this type of vaccination programme. Using a modelling system that focused on HPV transmission and vaccine coverage in England, they predicted an initial widening of the gap in cervical cancer incidence between whites and ethnic minorities in England, due to differential HPV vaccine uptake and herd immunity benefits within communities that lack miscegenation. Using this model, Johnson’s group predicted a 50% reduction in cervical cancer incidence occurring 20–30 years after initiation of their HPV vaccination programme and with aggressive HPV vaccination, zeroing out the gap in cervical cancer incidence between ethnic minorities and whites in 20 years [26]. In a subsequent commentary, Ginsburg and Paskett stress the need for improving vaccination rates among ethnic minorities, engaging governmental agencies to invest in cervical cancer prevention and control, and addressing the social inequities that drive cervical cancer incidence and mortality even in HICs like England [27].

In Australia where the incidence of cervical cancer among indigenous groups is twice that of non-indigenous groups, a recent national review reported no difference in vaccine coverage between indigenous and non-indigenous adolescents, but a completion coverage rate that favoured non-indigenous adolescents [28]. Jurisdiction differences in indigenous populations, and inaccurate status reporting of indigenous participants in the HPV vaccine programme present gaps that must be addressed to ensure equal prevention opportunities for all high-risk sub-groups. This is similar to what is seen among low income and ethnic minorities in the US where the initiating dose coverage is similar or higher among African Americans compared to white adolescents, but a decrease in course completion among African Americans is also present [29]. With both minority populations in the US and Australia sharing a disproportionate risk for developing cervical cancer, the disparity in completion rates may not be significant if one dose of the vaccine provides equivalent immunogenic impact, as has been suggested by Schiller and Lowy [30]. In the US, disparities in cervical cancer screening, resulting in a levelling off cervical cancer incidence and mortality was demonstrated by Kish and co-authors using a geospatial modelling system. Immigrants, African-Americans, Hispanics and older women, particularly those of lower economic status and those living in economically deprived and rural areas of the country were shown to be at greatest risk. To overcome these ethnic and geographic disparities, the authors have recommended increasing access to screening through community involvement, door-to-door visits and the use of HPV self-collection [31]. These efforts should additionally include accurate data collection on ethnic and vulnerable group uptake of HPV vaccination, to ensure that all subgroups are equally exposed to cervical cancer prevention strategies.

Countries that follow the Rwanda system, i.e., introducing national HPV vaccination campaigns, may be taking a good first step towards cervical cancer elimination. However, they should be aware that challenges exist. Strong government buy-in (including strong support from the First Lady and the Minister of Health), and advocacy, as well as partnerships with international and private sector organisations, made the Rwanda vaccine roll out possible. A 3-year cost-free provision of vaccines and concessional pricing for the future will help to provide sustainability of Rwanda’s primary prevention program [10]. A cervical cancer screening program using VIA and care HPV has been launched in Rwanda. Screening with VIA is still the most cost-effective and efficient method of cervical cancer screening in resource-poor countries, however, because it is highly subjective and dependent on training and continued supervision, sensitivity in detecting grade 2 or higher lesions has been reported anywhere from 7.7% to 82.6% compared to *care* HPV, a low-cost same day testing that has sensitivities above 70%. Screening for HPV offers a more objective approach, and an opportunity to increase screening coverage through self-collection [32, 33].

As we move forward to execute the recommendation made through the UN Global Joint Programme on Cervical Cancer Prevention and Control [34], we encounter two paths that countries can follow. First, HICs like Australia and the UK, both with high vaccine coverage (85% and >90%, respectively), can follow the data-driven modelling system set forth by Australia to achieve major reductions in incidence and mortality and ultimately elimination of cervical cancer within the next 10 years. Second, LICs can follow the lead of Rwanda and initiate mass prevention-driven vaccination projects while building their screening, diagnostic and therapy infrastructure and can still achieve the elimination of cancer over a 30–40 year period and within the 21st century.

## Conclusion

Cervical cancer is a preventable disease and we have all the tools to eliminate it. Almost 40 years ago, with global cooperation, small-pox was eliminated. The same effort is needed for this disease. Every 2 minutes a woman dies from cervical cancer; this undoubtedly makes cervical cancer elimination a matter of public health urgency. The cornerstone of cervical cancer elimination is screening and prevention. Each country must implement an elimination strategy that fits into its own culture and geographic landscape. It is incumbent on developed nations to assist the most vulnerable nations in the world where the incidence of cervical cancer is the highest, and the impact of screening and prevention campaigns will be greatest. HICs like the US that fall short in primary prevention should set the example to increase coverage to both its rural and urban communities by messaging the HPV vaccines as a cancer-prevention vaccine. LICs should work to improve screening using VIA with plans to transition to HPV with time. Most important, however, is that all nations insist with a common voice to lower the cost of HPV testing and vaccination so that all women globally would have access to these proven prevention and screening strategies.
